# The Self-Absorptive Trait of Dissociative Experience and Problematic Internet Use: A National Birth Cohort Study

**DOI:** 10.3390/ijerph182211848

**Published:** 2021-11-12

**Authors:** For-Wey Lung, Bih-Ching Shu

**Affiliations:** 1 Calo Psychiatric Center, Pingtung 92541, Taiwan; forwey@seed.net.tw; 2 Graduate Institute of Medical Science, National Defense Medical Center, Taipei 11490, Taiwan; 3 International Graduate Program of Education and Human Development, National Sun Yat-Sen University, Kaohsiung 80424, Taiwan; 4 Institute of Education, National Sun Yat-Sen University, Kaohsiung 80424, Taiwan; 5 Department of Nursing, Institute of Allied Health Sciences, College of Medicine, National Cheng Kung University, Tainan 70101, Taiwan

**Keywords:** Taiwan birth cohort study, problematic internet use, absorptive trait, Chinese Oxford happiness questionnaire

## Abstract

Functional and excessive use of internet are hard to distinguish from each other, and internet use can affect adolescents’ development of self-identity. The aim of our study was to investigate the associated relationships between the risk and protective factors for internet use, including parental monitoring, the absorptive dissociative trait, having been bullied, exercise, self-perceived depressive mood, and happiness of 12-year-old adolescents. The Taiwan Birth Cohort Study dataset, which used a national household probability sampling method and included 17,694 12-year-old adolescents, was used for this study. Our results showed that 5.3% of adolescents reported spending more than five hours online during school days. Additionally, adolescents that spent more than five hours online during school days tended to have a higher absorptive trait, perceived less care from mothers, were more likely to have been bullied, and expressed a higher level of depressed mood, which led to a lower level of perceived happiness. Adolescents that spent more than five hours online during school days, compared to those that spent less than an hour online, were more likely to have been bullied, which effected their level of happiness, showing that they may be a group of higher concern. Therefore, spending more than five hours per day online maybe a clinical prevention indicator for problematic internet use.

## 1. Introduction

Internet networking has transformed how people communicate, entertain, behave, and educate. However, along with the convenience that technology has brought, it has also brought growing adverse effects, such as pathological issues. Internet usage has been found to affect the brain and cognitive process [1]; this influence on “digital natives” is especially important. Adolescents are at a stage of development where they are searching for their identities and forming their self-image, along with developing executive function [2]. Internet use could interfere with these developments [2]. However, functional and excessive use of internet is hard to distinguish, and even in children (2 to 5.5 year-olds), 90.5% reported low to moderate screen use and 9.5% high-persistent screen use [3]. Internet addiction was proposed to be included in the fifth edition of the Diagnostic and Statistical Manual of Mental Disorders. Alongside this debate was a debate whether the diagnosis should be towards generalized internet use and/or potentially addictive activities that can be engaged on the internet (e.g., gaming, social media, pornography, information seeking, and shopping). The final DSM-5 introduced internet gambling disorder as a subtype of pathological gambling under the category of Substance-Related and Addictive Disorders.

Internet addiction has been reported to be a public health concern in both China and South Korea [4]. In Germany, a 1.16% prevalence was found for internet gaming disorder in adolescents [5]; however, a much higher prevalence of 6% was found in Korea [6]. Internet gaming disorder has been found to be related to internalizing and externalizing problems in teenagers, including depression, anxiety, impulsivity, and aggression [6]. Besides using the internet for gaming, increased use of social media screen time has also been found to be a risk factor for depression and suicide [7], showing that excessive internet use can have a damaging effect on mental health.

Besides internet use, bullying has also been found to affect the psychological well-being of adolescents [8]. Although internet use has its downside, other studies have found that social media use can boost wellbeing [9]. Shaw and Grant proposed that people with low self-esteem are more likely to decrease loneliness, modify their mood, and seek social support online [10]. Kurniasanti et al. also proposed that internet gaming can be used as a method of mood modification to adjust mood, get away from reality, and overcome depression [2]. Adolescents who experience bullying in real life may seek social support online; however, over a third of children who have experienced cyberbullying also reported having experienced bullying in real life [11]. Internet usage is a coping strategy to avoid concentrating on bullying and trauma experiences [12] and has also been found to be a mediating factor between bullying victimization and suicidal ideation [13]. Nevertheless, whether adolescents seek social support online because they have experienced interpersonal difficulties in real life, or vice versa, is yet to be determined.

In addition to the possibility of seeking social support, the individual’s own traits may also increase their risk to becoming addicted online. Schimmenti and Caretti proposed that the internet is a median for non-integrated self-states in individuals to express dissociative or non-integrated states of mind that have not been processed on a cognitive level or are not emotionally regulated in offline relationships [14]. A previous study found an association between dissociative experiences and internet addiction [15]. Another study further found that individuals with problematic internet use (PIU) and dissociation had more severe PIU and mental health problems, compared to those with PIU, but without dissociation [16]. The absorptive trait within the dissociative experience is the tendency to involuntary narrow one’s attention to the point of being oblivious to the environment [17], which has also been found to be associated with smartphone addiction in adolescents [18]. Therefore, the absorptive dissociative trait is also included in our investigation as a potential risk factor for internet addiction.

Besides the risk factors for internet addiction, we were also interested in protective factors that may prevent internet usage from influencing the daily activity and social interaction of adolescents. Family guidance and monitoring can prevent adolescents’ involvement in problematic and hazardous behaviors. On the other hand, families with high levels of conflict and dysfunction were strong predictors for internet addiction [19].

Besides family, physical exercise can also improve psychological adjustment and wellbeing [20]. A Korean study found adolescents that were physically active were more satisfied with their sleep, felts less stressed, and were less likely to be PIUers [21]. Similarly, among university students in the U.S., frequency of internet use was found to be associated with a lower grade point average, less frequent exercise, and greater perceived stress [22]. This shows that exercise is negatively correlated with PIU; thus, it may act as a protective factor for PIU. Exercise along with cognitive behavioral therapy has been found to improve symptoms of patients with depression [23]. Therefore, whether parental monitoring and exercise are resilience factors to internet addiction will also be investigated in our study.

The Taiwan Birth Cohort pilot study found that 12-year-old adolescents who spend more than five hours online during days without school were at a higher risk of deliberate self-harm and perceived a lower level of happiness [24]. Using a national birth cohort dataset which was collected two years later than the pilot study, the aim of this study was to investigate the risk and protective factors of internet use, including the associated relationship of parental monitoring, the absorptive dissociative trait, bullying, exercise, internet use, self-perceived depressive mood, and happiness of 12-year-old adolescents.

## 2. Materials and Methods

### 2.1. Participants

This study used the Taiwan Birth Cohort Study (TBCS) 12-year-old dataset. Aiming at building a sample representative of the children in Taiwan, the TBCS used the household probability sampling method. Babies born in the year 2005 were randomly selected with no exclusion criteria [25]. A two-stage stratified random sampling was used, at the first stage, the primary sampling unit was city and town [25]. Eighty-five townships were systematic randomly selected by grouping all 369 townships in Taiwan into 12 strata, according to four levels of size of the settlement in which the subjects were resident and three levels of total fertility rate. In the second stage, newborns were proportionally selected according to the rate of births from the 85 selected settlements [25]. When the children were 6 months old, 21,248 babies and families were selected (11.7% selection rate) [25]. The 12-year-old dataset is the fifth stage dataset; 18,814 families agreed to participate, and of these, 17,694 (94.05%) adolescents completed the questionnaires. The protocol of the study was approved by the institutional review board of a teaching hospital. Written informed consent was obtained at each stage of the study after a detailed explanation of the study.

### 2.2. Materials

All factors analyzed in this study were self-reported by the adolescents.

**Absorptive trait**. The TBCS included three items from the Adolescent Dissociative Experience Scale [26]. “I get so wrapped up in watching TV, reading, or playing a video game that I don’t have any idea what’s going on around me.” “I am so good at lying and acting that I believe it myself.” “I can’t figure out if things really happened or if I only dreamed or thought about them.” Response choices ranged from 1 (“strongly agree”) to 4 (“strongly disagree”). After recoding the responses, with a higher total score implying a higher absorptive trait, the Chronbach’s alpha of the three items resulted in 0.559.

**Happiness**. The self-perceived happiness of the adolescents was measured using the seven item Chinese Oxford Happiness Questionnaire, which has shown good psychometric properties in community adolescents in Taiwan [27]. Higher scores implied better perceived happiness.

**Internet use**. The TBCS asked two questions: “How many hours do you spend online during school days?”, and “How many hours do you spend online on days when you don’t have to go to school?” In addition to the continuous variable derived from these questions, the hours adolescents spent online were further dichotomized into those who spent less than an hour and those who spend more than five hours a day online. Twenge, Joiner, Rogers, and Martin found that adolescents who spent more than five hours a day on social media and smartphones had a 66% increased risk in suicide compared to those who spent less than an hour online [8].

**Exercise**. An item, “Do you exercise regularly?”, within the TBCS was used to measure adolescents’ exercising habits. Response choices were “Yes” = 1, and “No” = 0.

**Depressive state**. The item “I felt depressed” from the Center for Epidemiological Studies-Depression [28] was used to measure adolescents’ depressive state. Response choices were “never” = 1, “once in a while” = 2, “sometimes” = 3, “often” = 4, and “always” = 5.

**Parental monitoring**. Adolescents’ self-perceived level of parental monitoring was measured by asking the adolescents three questions: (1) “Does your mother (or the person who mainly takes care of you) know what you do on your free time?” (2) “Does your mother (or the person who mainly takes care of you) know who you normally hang out with (e.g., When you go out to play, exercise, shopping, do homework, etc.?” (3) “Does your mother (or the person who mainly takes care of you) know when you go to bed?”. These items were answered in a five-point Likert scale of “always”, “often”, “sometimes”, “once in a while”, or “never”. The ratings of these items were combined to form the parental monitoring factor, which has a Cronbach’s alpha of 0.702.

### 2.3. Statistical Analysis

The demographic distribution of the adolescents and parents were analyzed using Statistical Package for the Social Sciences (SPSS) 20.0 for Windows software (SPSS Inc., Chicago, IL, USA). Bayesian analysis, a multiple imputation method based on item response theory accounting for multiple sources of correlation, was used to replace missing data.

The structural equation model (SEM) was used to investigate the associated relationship of internet use, dissociative absorption experiences, being bullied, and perceived level of happiness of these adolescents. The SEM was analyzed using the Analysis of a Moment Structures 7.0 statistical software package (SPSS Inc., Chicago, IL, USA). SEM models with a *p* value greater than 0.05, an adjusted goodness-of-fit index (AGFI) greater than 0.9, a root mean square error of approximation (RMSEA) less than 0.08, a comparative fit index (CFI) greater than 0.95, and a Tucker–Lewis index (TLI) greater than 0.95 imply that the null model approximates the real structure.

## 3. Results

The demographic distribution of the adolescents and parents is shown in Table 1. Results showed 75.7% teens reported never having been bullied, which means approximately a quarter of the children had ever experienced bullying. Adolescents report being online on average of 1.55 (standard deviation = 1.87) hours during school days, and 3.62 h on days without school. Additionally, 5.3% of the teens report going online more than five hours on school days, and 25.9% on days without school. The majority of the teens reported exercising on a regular basis (82.1%).

Two SEMs were analyzed to investigate the associated relationship of internet use, absorptive trait, bullying, depressive state, and level of happiness in teenagers, with parental monitoring and exercise being the resilience factors within the model. The first model included internet use as a continuous variable, and in the second model internet use was dichotomized into those who were online less than one hour or more than five hours a day (those who reported being online between one and five hours were excluded in this model).

**Figure 1:**
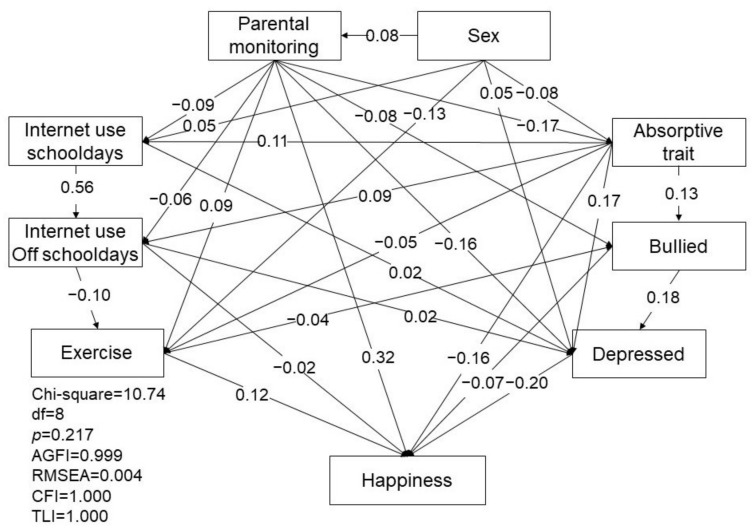
The path relationships among dissociative trait, bullying, internet use, depressive mood, and happiness in 12-year-olds. AGFI: adjusted goodness-of-fit; RMSEA: root mean square error of approximation; CFI: comparative fit index; TLI: comparative fit index.

The first SEM of the associated relationship of internet use (during school days and on days off school), absorptive trait, bullying, depressive state, parental monitoring, exercise, and level of happiness in teenagers is shown in Figure 1. The model resulted in a good fit, with a *p* value of 0.217 (greater than 0.05), AGFI of 0.999 (greater than 0.9), RMSEA of 0.004 (less than 0.08), CFI of 1.000 (greater than 0.95), and TLI of 1.000 (greater than 0.95). Factors which were associated with the teenagers’ time spent online included the teenagers’ sex, perceived parental monitoring, and the level of absorptive trait. Girls, lower parental monitoring and/or a higher absorptive trait were associated with more hours of internet use during school days (β = 0.05, *p* < 0.001; β = −0.09, *p* < 0.001; β = 0.11, *p* < 0.001). In a similar line, spending more hours online during school days, perceiving lower parental monitoring, and/or a higher absorptive trait were associated with more hours of internet use on days without school (β = 0.56, *p* < 0.001; β = −0.06, *p* < 0.001; β = 0.09, *p* < 0.001). Teenagers who spent more time online (both during school days and off school days) perceived lower parental monitoring, a higher absorptive trait, have been bullied, and/or were female, reported being more depressed (β = 0.02, *p* = 0.010; β = 0.02, *p* = 0.012; β = −0.06, *p* < 0.001; β = 0.17, *p* < 0.001; β = 0.18, *p* < 0.001; β = 0.05, *p* < 0.001). Factors associated with level of happiness included parental monitoring, absorptive trait, exercise, internet use during school days and on days without school, being bullied, and depressive state. Teens that perceived more parental monitoring, exercised more, had never been bullied, and had a lower absorptive trait and depressive level had a better overall level of happiness (β = 0.32, *p* < 0.001; β = 0.12, *p* < 0.001; β = −0.07, *p* < 0.001; β = −0.16, *p* < 0.001; β = −0.20, *p* < 0.001). Teens that spent more time online on days without school perceived a lower level of happiness (β = −0.02, *p* = 0.003).

**Figure 2:**
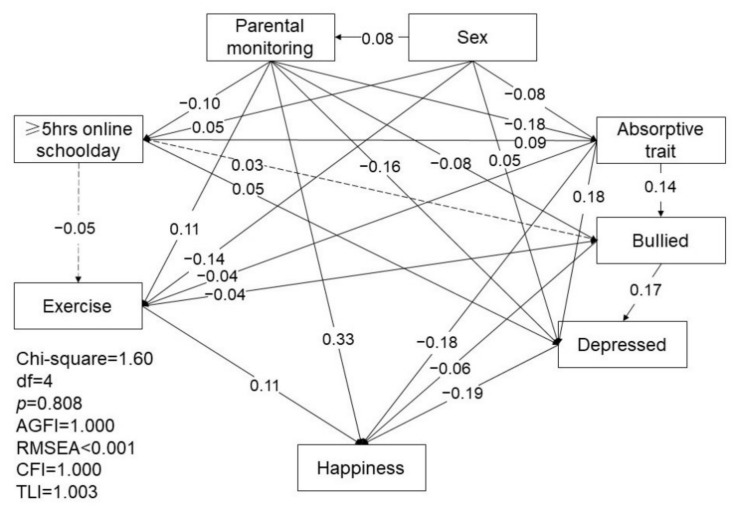
The path relationships of risk and protective factors for five hours of internet use on mood and happiness in 12-year-olds. “≥5 h online schoolday” dummy variable: 1 = spend ≥5 h online during schooldays, 0 = spend ≤1 h online during schooldays. AGFI: adjusted goodness-of-fit; RMSEA: root mean square error of approximation; CFI: comparative fit index; TLI: comparative fit index.

The second SEM investigated the associated differences of sex, parental monitoring, absorptive trait, exercise, internet use during school days, and depressive state in teens that spent more than five hours or less than an hour online during school days, as shown in Figure 2. The model resulted in a good fit, with a *p* value of 0.862, AGFI of 1.000, RMSEA of less than 0.001, CFI of 1.000, and TLI of 1.003. Similar to the results of the first figure, females, those who perceived lower parental monitoring, have been bullied, and/or had a higher absorptive trait were more likely to spend more than five hours online per day during school days (β = 0.05, *p* < 0.001; β = 0.03, *p* = 0.011; β = −0.10, *p* < 0.001; β = 0.09, *p* < 0.001). This model further found that teens who spend more than five hours online on school days were less likely to exercise and more likely to be depressed (β = −0.05, *p* < 0.001; β = 0.05, *p* < 0.001). Those who perceived higher parental monitoring, had a lower absorptive trait, had a lower frequency of being bullied, exercised more, and were less depressed reported a higher level of happiness (β = 0.33, *p* < 0.001; β = −0.18, *p* < 0.001; β = −0.06, *p* < 0.001; β = 0.11, *p* < 0.001; β = −0.19, *p* < 0.001).

## 4. Discussion

Our national birth cohort study found that among the 17,694 adolescents that participated, 5.3% reported spending more than five hours online during school days. Adolescents that spent more than five hours online during school days reported having a higher absorptive trait, perceived lower parental monitoring, were more likely to have been bullied, and expressed higher depressed mood, which led to a lower level of perceived happiness. Adolescents that spent more than five hours online during school days, compared to those that spent less than an hour online, were more likely to have been bullied which affects their level of happiness, showing that they may be a more pathological group, which may affect their occupational function (which for this group of students is being in school).

Approximately five percent of the teenagers in our study reported spending more than five hours online during school days. Although currently there is still no consistent set of criteria to define internet addiction, our study used the cutoff point of spending more than five hours online during school days and a 5.3% prevalence was found, which is within the range of 1.5% to 8.2% found in a review study [29].

With regards to bullying, approximately a quarter (24.7%) of the adolescents in our study reported to have been bullied; however, among teenagers who go online more than five hours a day, 31.15% reported having been bullied in school. The reported rate of bullying is similar to the 25.3% found in the Taiwan Birth Cohort pilot study (a cohort collected two years earlier than the Taiwan Birth Cohort Study) [30], which is similar to the 16.9% found in the United States [31]. Furthermore, our study found that teenagers that were bullied in school were more likely to be in this high-risk group for excessive internet use. Lam, Peng, Mai, and Jing found that recent stressful events as a risk factor for internet addiction [32], and Young proposed excessive internet use as a coping mechanism to avoid negative emotions and problems [33]. Internet use is a coping strategy to avoid concentrating on bullying and trauma experiences [12], and both substance and behavioral addictions can be conceptualized as a form of maladaptive self-regulatory strategy [34]. Shi et al. found a similar pathway relationship between bullying, internet addiction, and suicidal ideation [13]; With internet addiction being the mediating factor between bullying victimization, and suicidal ideation [13]. Therefore, adolescents who have been bullied may seek interpersonal connections by going online, attempting to decrease their negative emotions, but, however, increasing their risk for PIU.

In addition to the stressful event of being bullied, the personal characteristic of high absorptive dissociative trait also increases their risk of becoming addicted online. This is in line with previous studies which found dissociative experiences to be associated with internet addiction [14]. Furthermore, the absorptive trait within the dissociative experience have been found to be associated with smartphone addiction [18]. A meta-analysis also found “escape from self” to be an important intrapersonal risk factor for internet addiction [34]. Adolescents may experience great distress in the process of identify formation; those who are unable to overcome the stress in reality, may turn to the internet for a transient escape from real life and satisfaction from the virtual self, leading to internet addiction [35].

Besides the risk factor of absorptive trait and being bullied, we found parental monitoring and regular exercise were protective factors to being internet addicted. Parental monitoring and protection can decrease the adolescents’ motivation to participate in social networking and become internet addicted [36]. It also serves as a protective factor preventing teenagers from participating in problematic and harmful behaviors [37]. Systematic review also supports our results showing parental supervision and monitoring as protective factors for cyberbullying [38]. In contrast, a previous study found teens with internet addiction had a higher rate of conflict with parents compared to non-addicts [39]. Those adolescents who experience conflict at home or bullying in school may seek interpersonal connection and a sense of belonging online. Besides family monitoring, regular exercise was another protective factor. Exercise itself can boost mood and the psychological well-being of an individual, and a previous study also found those who were not addicted to the internet more frequently attended stress-releasing leisure activities compared to those who were addicted online [40].

As for the consequences of being addicted online, our study found that those who spent more time online were more likely to experience depressed mood and perceived lower level of happiness. The association between internet addiction and depressed mood have also been found in a previous study [7]. Twenge, Joiner, Rogers, and Martin found that adolescents who spent more time on new media screen activities (such as smartphone devices and social media) were more likely to report mental health issues, and those who reported to have spent more time on non-screen activities (such as exercise or in-person interaction) were less likely to report mental health issues [8]. Furthermore, the Taiwan Birth Cohort pilot study found that 12-year-old adolescents who spend more than five hours online during days without school were at a higher risk of deliberate self-harm at thirteen, as well as a perceived lower level of happiness [24]. The negative relationship between internet use and exercise was also found in our study; adolescents that spent more time online were less likely to exercise regularly. Furthermore, those that exercised regularly perceived higher level of happiness, and those who spent more time online perceived lower level of happiness.

A limitation of our study was that only the hours that adolescents spent online were collected, and no information regarding the behavioral consequences of internet addiction was collected. This is part of the constraint of using national birth cohort dataset; since different aspects associated with children’s development and health is collected, only limited information about each dimension can be obtained. The current proposed diagnostic criteria for internet addiction include the four components of: (1) excessive use, (2) withdrawal, including feelings of anger, anxiety, or depression when the access to a computer or smartphone is limited, (3) tolerance, including the need for more hours of use, and (4) adverse consequences, including fatigue, social isolation, or poor academic or occupational performances [4]. However, since there are still no official diagnostic criteria for internet addiction, no diagnostic consensus has been reached. Furthermore, only information on general use of internet was collected, while no information regarding habits of use was collected. Young and Cristiano have proposed that internet usage be separated into four categories: gaming, social media, pornography, and information seeking [41]. Further information regarding the purpose of internet use may provide us with more information regarding the different subtype of internet addiction and its effects. Another limitation of this study is that only the 12-year-old dataset of the TBCS was used; thus, it is a cross-sectional design. Further follow-up on the influence of bullying, parental monitoring, exercise, and these association with hours of internet use in this group of adolescents, will provide us with more information regarding the risk and protective factors of problematic internet use.

## 5. Conclusions

Our large national birth cohort study showed that those with higher absorptive dissociative trait, lower parental monitoring, and who have been bullied in school were more likely to spend more than five hours online during school days and expressed higher depressed mood and a lower level of perceived happiness. This result is in line with the Taiwan Birth Cohort pilot study (*n* = 1457), which found that adolescents who spend more than five hours online have an increased risk of deliberate self-harm and a lower level of happiness [24], showing that spending more than five hours online per day may be a clinical prevention indicator for problematic internet use. Since parental monitoring is a protective factor for internet addiction, parents should provide monitoring and guidance while respecting the autonomy of adolescents, thereby reducing the motivation of adolescents to become involved with social networking and risks for internet addiction [19,36]. Furthermore, adolescents who do not have adequate coping strategies when faced with stressful situations are more likely to choose avoidant methods, which increases their risk of becoming addicted. Exercise rehabilitation has been promoted by Kim to increase physical and mental health condition of those with internet addiction [42]. Therefore, it should be promoted to all adolescents, especially those in the high-risk group, such as those who spend more than five hours online per day, have been bullied, or have absorptive trait, to prevent them from being addicted online.

## Figures and Tables

**Table 1:** Demographic distribution of the adolescents and parents (*n* = 17,694).

Variable	*n* (%)
Child sex	
Boy	9246 (52.3)
Girl	8448 (47.7)
Have been bullied	
Always	171 (1.0)
Often	217 (1.2)
Sometimes	818 (4.6)
Once in a while	3087 (17.4)
Never	13,401 (75.7)
Online time on school days	
≤1 h	11,295 (63.8)
≥5 h	943 (5.3)
Online time on off school days	
≤1 h	5416 (30.6)
≥5 h	4576 (25.9)
Exercise regularly	14,518 (82.1)
Maternal education	
Illiterate	13 (0.1)
Elementary school	509 (2.9)
Middle school	1433 (8.1)
High school	6322 (35.7)
University/college	8391 (47.4)
Graduate school	1026 (5.8)
Paternal education:	
Illiterate	5 (0.0)
Elementary school	214 (1.2)
Middle school	1788 (10.1)
High school	6349 (35.9)
University/college	7473 (42.2)
Graduate school	1865 (10.5)
Variable (range)	Mean (SD)
Hours spend online	
School days	1.55 (1.87)
Days without school	3.62 (3.38)

## Data Availability

Taiwan Birth Cohort Study datasets can be accessed from the Taiwan Ministry of Health and Welfare, Bureau of Health Promotion https://dep.mohw.gov.tw/DOS/np-2500-113.html (accessed on 24 September 2021).
